# Laccase activity of the ascomycete fungus *Nectriella pironii* and innovative strategies for its production on leaf litter of an urban park

**DOI:** 10.1371/journal.pone.0231453

**Published:** 2020-04-16

**Authors:** Aleksandra Góralczyk-Bińkowska, Anna Jasińska, Andrzej Długoński, Przemysław Płociński, Jerzy Długoński

**Affiliations:** 1 Department of Industrial Microbiology and Biotechnology, Faculty of Biology and Environmental Protection, University of Lodz, Lodz, Poland; 2 Department of Environmental Engineering, Faculty of Biology and Environmental Sciences, Cardinal Stefan Wyszynski University in Warsaw, Warsaw, Poland; 3 Institute of Medical Biology of Polish Academy of Sciences, Lodz, Poland; Tallinn University of Technology, ESTONIA

## Abstract

A laccase-producing ascomycete fungus was isolated from soil collected around the premises of a textile dye factory and identified as *Nectriella pironii*. Efficient laccase production was achieved *via* the synergistic action of 1 mM copper sulfate and ferulic acid. Extracts of rapeseed oil cake, grass hay, and leaf litter collected in a pocket urban park were used for enzyme production. The highest laccase activity (3,330 U/L) was observed in the culture grown on the leaf litter extract. This is the first report on biosynthesis of laccase by *N*. *pironii*. This is also the first study on utilization of naturally fallen park leaves as a substrate for fungal laccase production. The extracellular enzyme possessing laccase activity was purified to homogeneity by ion-exchange and gel filtration chromatographic techniques. The amino acid sequence of the protein revealed highest similarity to the laccase enzyme produced by *Stachybotrys chartarum*—and considerable homology to those produced by other fungal species. The purified laccase possessed a molecular mass of 50 kDa. The enzyme had an optimum pH of 2.0 or 6.0 and retained more than 50% of residual activity after 3 hours of incubation at pH 3.0–10.6 or 4.0–9.0 when 2,2′-azino-bis(3-ethylbenzothiazoline-6-sulphonic acid or 2,6-dimethoxyphenol, respectively, were used. Dithiothreitol, β-mercaptoethanol, and sodium azide at 1 mM concentration strongly inhibited the laccase activity, while in the presence of 50 mM urea, the enzyme was found to retain 25% of its activity. The laccase was able to decolorize more than 80% of Indigo Carmine, Remazol Brilliant Blue R, Reactive Orange 16, and Acid Red 27 dyes within 1 h. The possibility of leaf litter use for the production of the laccase enzyme from *N*. *pironii* (IM 6443), exhibiting high pH stability and degradative potential, makes it a promising tool for use in different environmental and industrial operations.

## Introduction

Laccases (EC 1.10.3.2) are a group of copper-containing enzymes (multicopper oxidases, MCOs) commonly found in plants, bacteria, fungi, and insects. Fungal laccases are involved in sporulation, pigment production, fruiting body formation, and plant pathogenesis. These copper-containing enzymes catalyze the oxidation of a variety of phenolic and nonphenolic substrates with a simultaneous reduction of molecular oxygen to water [1, 2].

Due to their ability to oxidize a wide range of substrates without the requirement of any coenzyme factors and high efficiency, laccases have found application in many industries, medicine, environment protection, and various biotechnological processes [1, 3]. Laccases have been successfully used in delignification, paper pulping, and pretreatment of biomass for the production of biofuel [4, 5, 6]. They are also widely applied in wastewater treatment, degradation of xenobiotics, and as dye decolorizing agents. Water released from textile industries is polluted with dyes and is reported to be one of the top ten contaminating sources of water bodies. Due to the fact that traditional processes do not remove all dyes and are expensive, laccases provide a safe and efficient alternative for decolorizing and detoxification of dyes before their discharge into the environment [7, 8].

On the other hand, the absence of efficient expression system and high production costs hinder the large-scale production of laccases. Therefore, there is a need to identify new sources of laccases and find new methods of obtaining the enzyme in a rapid and economical way. Increase of enzyme biosynthesis through modification of the culture medium composition, changing the processing conditions, and supplementation with chemical inducers, such as metal ions and aromatic compounds, is a well-known solution for this problem. However, few studies indicate synergistic stimulation of laccase production by metal ions and aromatic compounds [9, 10]. Also, utilizing bio-waste to obtain an enzyme can reduce production cost while generating higher concentrations of products [11]. Till date, especially agricultural waste such as fruit peels, cereal bran, and straw or oil cakes have been successfully used for laccase production. Such residues contain polysaccharides and phenolic compounds that can stimulate both the fungal growth and subsequent laccase production [12]. Our previous study indicated rapeseed oil cake, hay, and sawdust as convenient substrates for laccase production by *Myrothecium roridum* [13]. High activity of the produced enzyme can be attributed to the presence of high content of reducing sugars and phenolic compounds, such as gallic or ferulic acid, in the media. Phenolic compounds are one of the plant secondary metabolites that play important roles in providing disease resistance, protection against pests, and species dissemination. Their presence has been reported in the leaves of trees such as *Betula pendula* [14], *Salix* sp. [15] and *Aesculus* sp. [16, 17] as well as the leaf litter of Central Europe’s forests [18]. According to Chua and Hidayathulla [19], fallen senescent leaves contain more amounts of phenolic compounds compared to green leaves. Public green space including urban forests and parks form a significant portion of Central Europe cities, e.g. in Łódź [20] and Leipzig [21]. Nevertheless, the data with regard to the possibility of using naturally fallen leaves as a substrate for microbial enzymes production are scarce. Bio-waste collected from the recreational parks of urban agglomerations is mainly used for compost manufacture and rarely for energy production in biogas stations or incinerators [22]. The utilization of leaf litter (collected and gathered from urban parks during every autumn) for laccase biosynthesis could result in a more economical production process and additionally encourage self-sufficiency of the greenery.

This study was aimed to estimate possibility of leaf litter application for the newly identified fungal laccase with biodegradative potential. To the best of our knowledge, extract of leaf litter collected in an urban park during autumn season was for the first time utilized as a substrate for fungal laccase production. Subsequently, the characterization of the newly identified enzyme has been performed. It included molecular weight determination, pH stability, and effect of inhibitors on the laccase activity. The capacity of the enzyme to decolorize the dyes was also determined.

## Materials and methods

### Chemicals

The compounds 2,2′-azino-bis(3-ethylbenzothiazoline-6-sulphonic acid) (ABTS), 2,6-dimethoxyphenol (2,6-DMP), sodium azide (NaN_3_), kojic acid, caffeic acid, ferulic acid, guaiacol, catechol, copper sulfate, manganese sulfate, syringaldazine, Indigo Carmine (IC), Remazol Brilliant Blue R (RBBR), Reactive Orange 16 (RO 16), and Amaranth (Acid Red 27 (AR 27)) were purchased from Sigma-Aldrich (high purity reagent grade).

### Bio-waste components of fungal media

Rapeseed oil cake commonly used in pig nutrition [23] and previously utilized by Jasińska et al. [13] for laccase production by *M*. *roridum* IM 6482 was obtained from BIO-TECH Ltd (Gorczyn, Poland).

Commercial grass hay manufactured for rabbits and other small animals and previously used in our former study on laccase biosynthesis by *M*. *roridum* IM 6482 [13] was supplied by Eko-Sianko (Łask, Poland).

**Fig 1:**
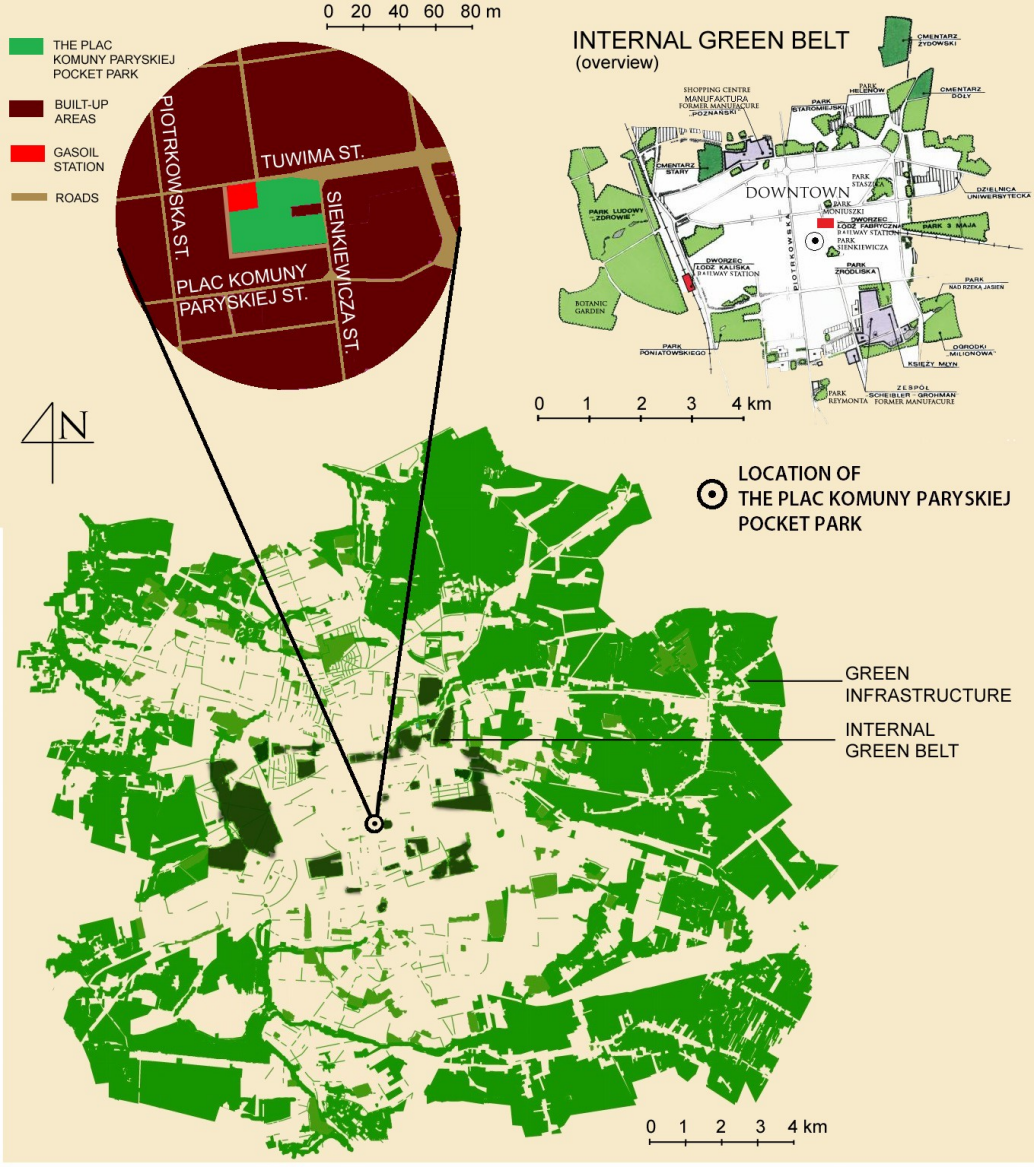
Distribution of the urban green spaces in Łódź.

Naturally fallen foliage of trees (*Aesculus hippocastanum* L., *Populus simonii* “Fastigiata”) and bushes (*Ribes alpinum* L.) was collected in the area of Plac Komuny Paryskiej pocket park (Łódź, Poland) with the surface area of 0.2 hectares at the end of autumn. This area is located within the internal Green Belt of Łódź (Fig 1) and is a valuable element of Łódź Green Infrastructure [22, 24, 25].

### Media preparation for laccase production by fungal isolates

In this study, rapeseed press cake extract, grass hay, and leaf litter were used as substrates for the media preparation according to the protocol described by Atlas [26] with slight modifications. Before media preparation leaves have been washed with distilled water to remove soil residue and other contaminants. Rapeseed press cake extract (50 g), dry hay (20 g), dry leaf litter (25 g) were added separately to deionized water, and the final volume was made up to 1.0 L and mixed thoroughly. After boiling, solid residues have been removed *via* the filtration through 0.22 μm pore membrane filters and the extract was autoclaved at 117ºC for 20 min. The pH of the extract was adjusted to 6.8 and supplemented with 2% (w/v) glucose and 1.0% (w/v) neopeptone. In the next step, 1 mM CuSO_4_ was added to induce laccase production and the media were inoculated with 10% inoculum. The submerged cultures were prepared in Erlenmeyer flasks and incubated on a rotary shaker (120 rpm) at 28°C for 5 days.

### Isolation of filamentous fungi from a postindustrial urban area and screening for laccase activity

Twenty-six fungal strains used in this study had been previously isolated from soil samples collected from the postindustrial textile green space of the Łódź Metropolitan Area (Poland) and stored at the strain collection center of the Department of Industrial Microbiology and Biotechnology, University of Łódź. The fungal strains were maintained on ZT slants [27] at 4°C and regularly transferred.

### Qualitative analysis

Primary screening of filamentous fungi for laccase activity was carried out by inoculation of 1 cm diameter of mycelium from each strain on a Kirk and Farell solid medium containing additionally 1 mM ABTS or 1 mM 2,6-DMP and then incubated at 28°C. The medium consisted of (g/L): glucose, 2.0; ammonium tartrate, 2.0; malt extract, 2.0; KH_2_PO_4_, 0.26; Na_2_HPO_4_, 0.26; MgSO_4_·7H_2_O, 0.5; agar, 2.0; ABTS, 0.35; CuSO_4_·5H_2_O, 10 × 10^−3^; CaCl_2_·2H_2_O, 6.6 ×10^−3^; FeSO_4_, 5 × 10^−3^; ZnSO_4_·7H_2_O, 0.5 × 10^−3^; Na_2_MoO_4_, 0.02 × 10^−3^; MnCl_2_·4H_2_O, 0.09 × 10^−3^; and H_3_BO_3_, 0.07 × 10^−3^, pH 5.5 [28]. The formation of a dark green halo in the plates supplemented with ABTS and an orange halo in the plates supplemented with DMP indicated a positive laccase secretion. The diameters of the halo zones and the mycelium were measured at regular intervals of time.

### Quantitative analysis

Quantitative examination of the fungal isolates that showed a positive laccase activity was carried out under submerged cultivation conditions. Inoculum (15 mL of 24-hour-old fungal culture) was introduced into 500 mL Erlenmeyer flasks containing 135 mL of modified Czapek-Dox medium composed of (g/l): yeast extract, 3.0; KH_2_PO_4_, 1.0; KCl, 0.5; MgSO_4_·7 H_2_O, 0.5; FeSO_4_·7 H_2_O, 0.01; and glucose, 7.5, pH 6.8. The flasks were incubated on a rotary shaker (120 rpm) at 28°C. Sampling was done at regular intervals for determining the laccase activity and the whole experiment was carried out in triplicate.

### Taxonomic identification of the IM 6443 fungal strain (DNA extraction, PCR amplification, and sequence data analysis)

Taxonomic identification was done by RDLS Heritage Ltd (Warsaw, Poland). Fungal genomic DNA was isolated using an Extract Me Genomic DNA Kit (Blirt S.A.). The gene fragment present in the 5.8S rRNA ITS region was amplified with the use of a Thermal Cycler (BioRad) using primer sets ITS1-F and ITS4-R. The PCR reaction was performed under the following conditions: initial denaturation (95°C for 3 min) and then denaturation (34 amplification cycles at 95°C for 30 s), annealing (54°C for 30 s), primer extension (72°C for 90 s), and final extension at 72°C for 10 min. Sequencing reactions were performed using a BigDye Terminator v3.1 Cycle Sequencing Kit (Thermo Fischer). The obtained sequence was compared using a NCBI BLAST similarity search tool. The sequence was analyzed phylogenetically by comparing with closely related sequences of reference organisms from the BLAST network service followed by multiple sequence alignment by using the algorithm MUSCLE 3.7 [29].

### Optimization of the laccase production

Fungal spores produced by 10-day-old cultures on ZT slants were used for the preparation of precultures in 25 mL of WHI medium [30] using 100 mL Erlenmeyer flasks. The incubation was conducted at 28°C on a rotary shaker (120 rpm). After 24 h, the precultures were transferred to fresh WHI medium in the ratio 1:4 and cultivated for subsequent 24 h. Next, 3 mL of the preculture was introduced into 27 mL of Sabouraud dextrose broth liquid medium (BioMaxima) supplemented with glucose (2%). Six different inducers (ABTS, 2,6-DMP, caffeic acid, ferulic acid, copper sulfate, and manganese sulfate) were added independently to Sabouraud fungal growth medium. The control flask was devoid of any inducer compound. The fungal isolates were incubated at 28°C for 6 days. Furthermore, the effect of combining copper ions with other inducers was evaluated. Sampling was done at regular intervals for measuring the laccase activity, and all the experiments were carried out in triplicate.

### Assay for determining laccase activity and protein concentration

The laccase activity was determined by using ABTS as substrate and measuring the absorbance change caused by the action of enzyme on the substrate within 1 min using a Specord 200 spectrophotometer (Analytic Jena, Germany) and WinASPECT PLUS software [31]. Molar absorption coefficient (ε) for ABTS at 420 nm and for DMP at 470 nm was 36,000 M^-1^ cm^-1^ and 14,800 M^-1^ cm^-1^ [32]. The reaction mixture contained 250 μL of 10 mM ABTS or DMP, 740 μL of Mc Ilvaine Buffer (composed of 0.2 M dipotassium hydrogen phosphate and 0.1 M citric acid, buffered to pH 4.5), and 10 μL of enzyme extract (Jasińska et al. [33]. One unit of laccase activity (U) was defined as the concentration of the enzyme required to oxidize 1 μM of substrate per minute.

Enzyme activity was calculated according to the formula adopted from Leonowicz and Grzywnowicz [34] with slight modifications:

$$Laccase\;activity\;\left[U/L\right]=(\Delta Abs\times V\times 10^{6})/\left(36,000\times Ve\times \Delta t\right),$$ (1)

where ΔAbs is the difference in absorbance values at Abs_60_ and Abs_0_;

V is the total volume of the sample [mL]; Ve is the volume of the enzyme [mL]; and Δt is the time [min].

The protein concentration was determined using the BCA test (bicinchoninic acid assay) according to the Pierce^™^ BCA Protein Assay Kit protocol (Thermo Fisher Scientific).

### Protein isolation and purification

The crude laccase enzyme was precipitated from the culture media using ammonium sulfate precipitation technique. Briefly, the fungal culture was centrifuged at 8,500 × g for 15 min at 4°C and the resulting supernatant was collected. Solid (NH_4_)_2_SO_4_ up to 60% saturation was added slowly to the supernatant and incubated at 4°C overnight with constant stirring. The precipitated proteins were sedimented by centrifuging at 8,500 × g for 15 min at 4°C, and the obtained pellet was resuspended in 100 mL of 0.05 M Tris buffer at pH 7.5. The enzyme solution was then concentrated using a 30 kDa ultrafiltration membrane (Amicon Ultra-15; Merck Millipore, USA) and purified on an FPLC System (ÄKTA Start; GE Healthcare). The buffered protein solution was first run on an ion-exchange HiTrap Q FF column (GE Healthcare) equilibrated with 0.05 M NaCl in 0.05 M Tris buffer at pH 7.5 and eluted in a linear gradient of salt at a flow rate of 2.5 mL/min. Both the wash and elution fractions were analyzed for laccase activity, as described in the previous section. Fractions with the highest laccase activity were pooled, concentrated by ultrafiltration, loaded onto a gel filtration HiPrep 16/60 Sephacryl S-200 column (GE Healthcare), and eluted at a flow rate of 1.0 mL/min. The resulting fractions containing purified laccase were pooled, concentrated, and used for further analyses.

### Mass spectrometry analysis of the purified protein

Purified laccase was resolved on a native 12% Tris-glycine polyacrylamide gel. The gel was then developed as a zymogram using the ABTS solution, which was formulated exactly similar to that used for the laccase activity testing. A single protein band showing strong positive ABTS staining was excised from the gel and used for mass spectrometry analysis. The gel slice was processed by following the in-gel trypsin digestion protocol, as described in detail by Shevchenko et al. [35]. The resulting tryptic peptides were next analyzed using the ion source of the Q Exactive^™^ Hybrid Quadrupole-Orbitrap Mass Spectrometer (Thermo Electron Corp) coupled to a nano-HPLC system fitted with a RP-18 column (Waters). The mass spectrometer was operated in a data-dependent mode with a selected mass range of 300–2000 mass/charge (m/z). The raw result files were processed directly using the MaxQuant v.1.6.3.4 or PEAKS X Studio software. Initially, the fragmentation spectra were searched against a user-defined database, created by fetching the FASTA records for all proteins annotated as “multicopper oxidase” from the NCBI protein database. The secondary database search was performed against a user-defined database created by fetching 1000 proteins from NCBI BLAST, showing the best sequence similarity to the primary multicopper oxidase identified in the initial search. The PEAKS X Studio database search was performed with *de novo* identified peptide set to confirm the protein sequence similarity between the already annotated and the newly characterized proteins.

### Laccase characterization

The pH-dependent activity of the purified enzyme was studied using ABTS (10 mM) at 420 nm and 2,6-DMP (10 mM) at 470 nm as substrates dissolved in the Mc Ilvaine buffer (pH 2.2–8.0) and Glycine-NaOH buffer (pH 8.6–10.6). The laccase activity after incubating the enzyme at 4°C for 3 h at varying pH values was estimated to determine the optimal pH at which the purified laccase showed maximum stability.

The inhibition effect of β-mercaptoethanol (β-ME), dithiothreitol (DTT), ethylenediaminetetraacetic acid (EDTA), kojic acid, sodium dodecyl sulphate (SDS), NaN_3_, and urea on the purified laccase activity was studied in Mc Ilvaine buffer at pH 3.0 using 10 mM ABTS as a substrate. For control measurement, the enzyme activity was determined without any inhibitor. The inhibitors were premixed with the enzyme to acquire a final concentration from 0.1 to 500 mM and incubated for 3 h at room temperature. As far as possible, the STRENDA guidelines were included in the description of the enzyme activity assays [36].

### Decolorization studies

The capacity of purified laccase to decolorize IC, RBBR, RO 16, and AR 27 dyes was determined spectrophotometrically by measuring the decrease in absorbance at 611, 593, 521, and 499 nm, respectively. The reaction mixture contained 25 mg/L of dye dissolved in Mc Ilvaine buffer (pH 3.0) and 1 U/mL of laccase. The mixture was incubated at room temperature for 60 min.

The percentage of decolorization was calculated as follows:

$$D\;\left[\%\right]=\left[100\times \left(A_{0}–A_{t}/A_{0}\right)\right],$$ (2)

where A_0_ is the absorbance of the abiotic control, A_t_ is the absorbance of culture supernatant, and D is the decolorization rate.

### Statistical analysis

The obtained results were presented as the mean ± standard error of the mean (SEM) of three independent samples. Student’s *t*-test was used to determine the statistical significance of differences between the mean values. P-value ≤ 0.05 was considered to indicate statistical significance. Statistical analyses were performed using Excel 2007 (Microsoft Corporation, USA).

## Results and discussion

### Screening of laccase-producing fungi

Due to their ability to oxidize both phenolic and nonphenolic compounds, laccases have been drawing the attention of researchers for the last few decades. Screening and selection of promising laccase producers from nature, followed by optimization of culture conditions, provide an effective approach to obtain organisms which have increased capacity to synthesize laccase.

**Fig 2:**
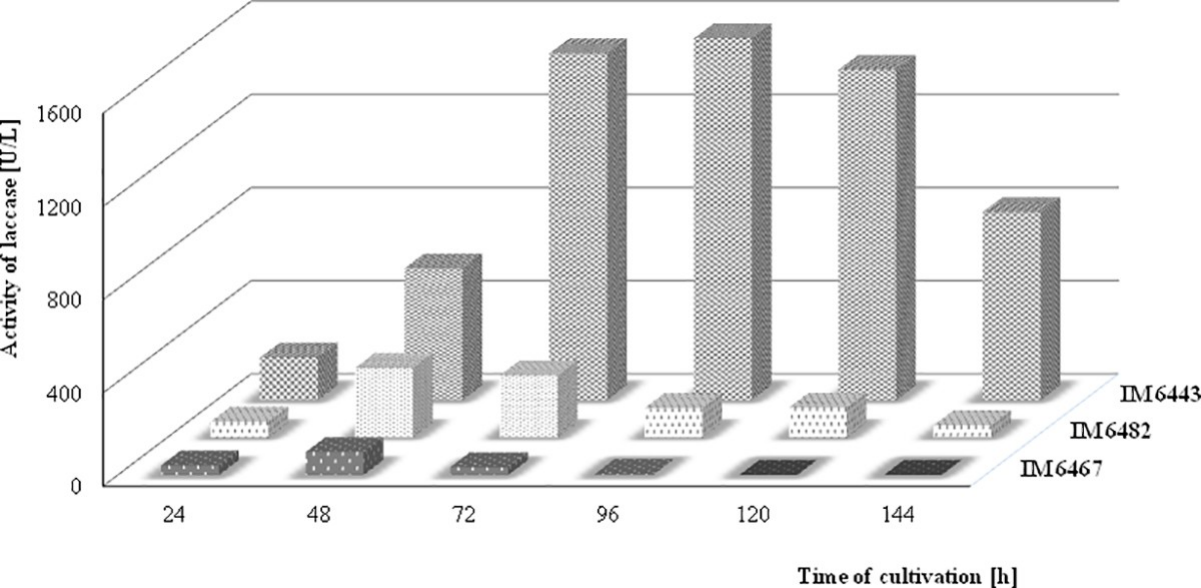
Time course of laccase production during IM 6443, IM 6467, and IM 6482 cultivation in modified Czapek-Dox medium containing 0.75% of glucose and 3.00% of yeast extract.

In this study, 26 fungal cultures were isolated from a postindustrial textile green area and tested for laccase production on the medium containing phenolic as well as nonphenolic substrates (Table 1). Fungal cultures were checked for zone formation on agar plates containing ABTS or DMP, which are considered as standard substrates for laccase [2]. This allowed for the indication of laccase-producing fungi. Among all the tested isolates, strains marked as IM 6443, IM 6467, and IM 6482 formed a dark green halo in ABTS-supplemented plates, while 2,6-DMP oxidation expressed by the formation of an orange halo was shown by the strains IM 6443 and IM 6482. Screening of laccase-producing microorganisms on solid media containing coloured indicator compounds is known to enable quick visual detection of laccase production; however liquid cultivations are necessary for the measurement of enzyme activity [9]. The laccase activity of the tested strains was estimated over the period of 6 days at regular intervals of 24 h under submerged fermentation conditions (Fig 2). Among the isolates, the strain IM 6443 was observed to show the highest laccase activity (about 1,600 U/L on 4th day) and hence was selected for further experimental work and identification.

**Table 1:** Qualitative screening for laccase positive isolates using ABTS and 2,6-DMP after 4 days of cultivation.

Strain number	ABTS		2,6-DMP	
	Colony diameter [mm]	Oxidation	Colony diameter [mm]	Oxidation
6440	45	-	50	-
**6443**	22	+++	30	+++
6448	35	-	42	-
6449	70	-	65	-
6451	22	-	20	-
6452	30	-	30	-
6456	30	-	25	-
6457	40	-	35	+
6459	37	-	24	-
6460	37	-	30	-
6462	60	-	65	-
6463	70	-	70	-
6464	35	-	30	-
**6467**	45	++	40	+/-
6470	22	-	42	-
6473	58	-	40	-
6474	39	-	35	-
6480	40	-	45	-
6481	60	-	55	-
**6482**	20	+++	25	+++
6485	30	-	35	-
6486	27	-	30	+
6487	78	-	75	-
6488	78	-	65	-
6490	32	-	40	-
6493	20	-	20	-

Activity: +++ high; ++ medium; + low; +/- ambiguous;—none

The molecular identification based on PCR amplification of the ITS r-DNA gene was carried out using the ITS1 and ITS4 primers. The phylogenetic analysis revealed that the IM 6443 strain showed 93% agreement with the *Nectriella pironii* strain CBS 264.80 (MH861261.1). The phylogenetic tree is presented in (S1 Fig).

### Optimization of the laccase production

**Fig 3:**
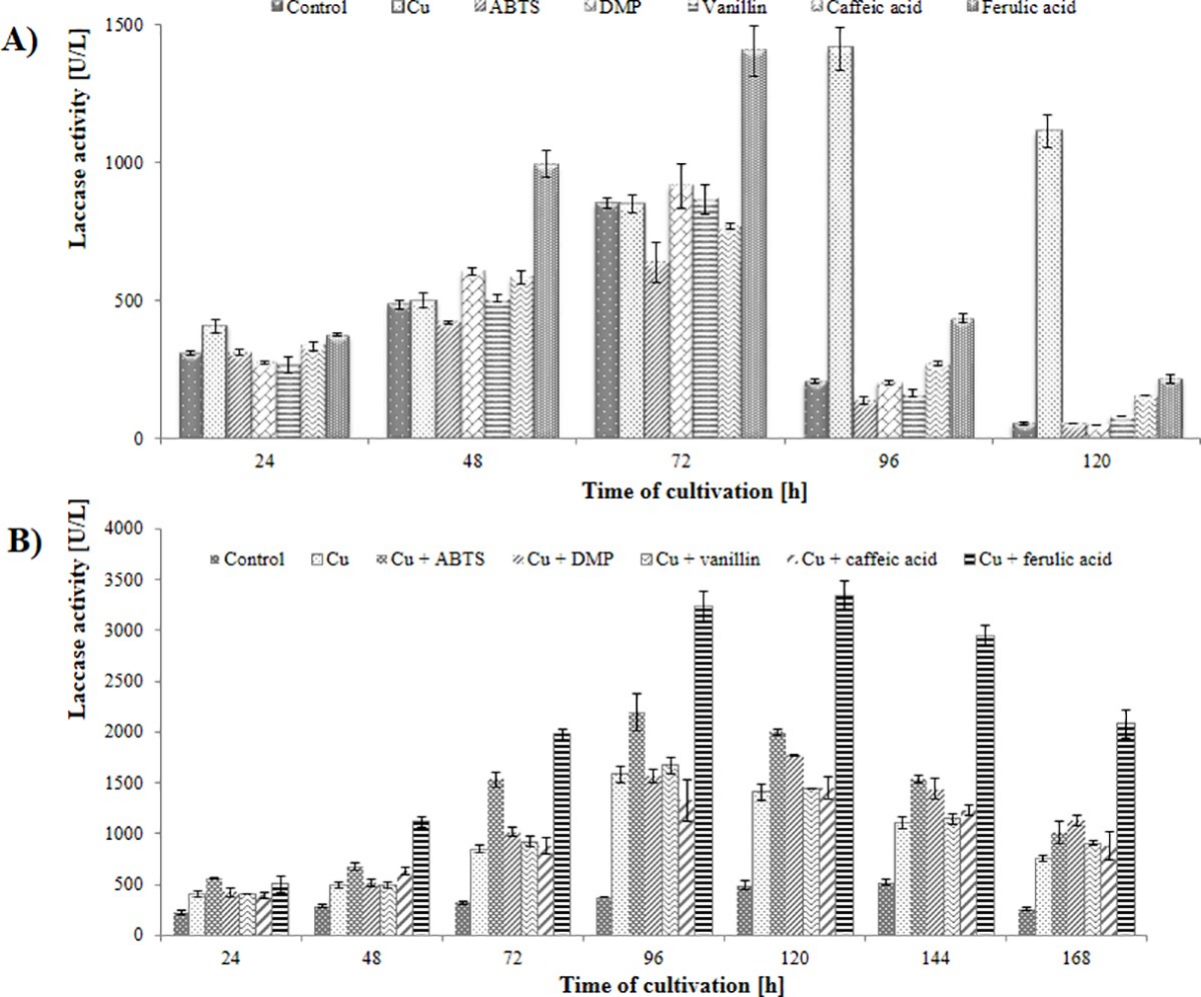
Laccase production by *Nectriella pironii* IM 6443 during the cultivation in Sabouraud medium supplemented with inducers (A) or their combination (B).

Large-scale applications of laccases are limited by the cost and efficiency of these enzymes [37, 38, 39]. Efforts have been made to produce large amounts of laccases at lower costs with the use of recombinant organisms or screening for naturally available hypersecretory strains. To enhance laccase production, different strategies can also be applied, such as optimization of culture conditions or use of lignocellulose materials as substrates [10, 13, 40]. Increased secretion of laccase by fungi when cultured in media supplemented with copper or different aromatic compounds has been well documented [41, 42]. In this study, the effect of ABTS, DMP, caffeic acid, ferulic acid, copper sulfate, and manganese sulfate on laccase secretion by *N*. *pironii* IM 6443 was determined. The induction of the enzyme in the cultures growing in Czapek-Dox medium was observed only in the presence of copper ions (data not shown), so further study was performed using Sabouraud medium. All the tested inducers increased the production of the enzyme (Fig 3A). Among them, the highest laccase induction was observed in the cultures enriched with copper sulfate and ferulic acid. After cultivating *N*. *pironii* IM 6443 in media containing these additives for 96 and 72 h, the enzyme activity reached 1,412 and 1,405 U/L, respectively, and was almost five fold higher than in the control sample cultivated in Sabuoraud medium. Copper has been reported to be a strong inducer of laccase in many fungal species, including *Aspergillus flavus* [43], *M*. *roridum* [44], and *Peniophora* sp. [45]. Copper not only regulates laccase gene expression but also positively affects the activity and stability of the enzyme by exerting an inhibitory effect on the activity of extracellular proteases [46]. On the other hand, the increased production of laccase in the presence of phenolic compounds may be a defensive reaction of the microorganism to oxidative stress, since these compounds are known to induce stress [47]. The surface of fungal hyphae contains specific receptors for small phenolic compounds that stimulate *de novo* synthesis of laccase [46]. Interestingly, in the present study, the combined supplementation of *N*. *pironii* IM 6443 cultures with copper sulfate and ferulic acid led to a significant increase in the production of laccase as compared to the cultures containing these compounds separately (Fig 3B). Activity of laccase determined after 120 hours of cultivation of the fungus in the media containing both mentioned inducers reached 3,347 U/L. The effect of the simultaneous use of copper and an aromatic compound on the laccase gene transcription was evaluated by Yang et al. [48]. They proved that copper ions and syringic acid exhibit a positive synergistic effect on the laccase production and laccase gene transcription in *Trametes velutina*. However, this is the first attempt involving simultaneous application of copper and ferulic acid for increasing laccase production.

**Fig 4:**
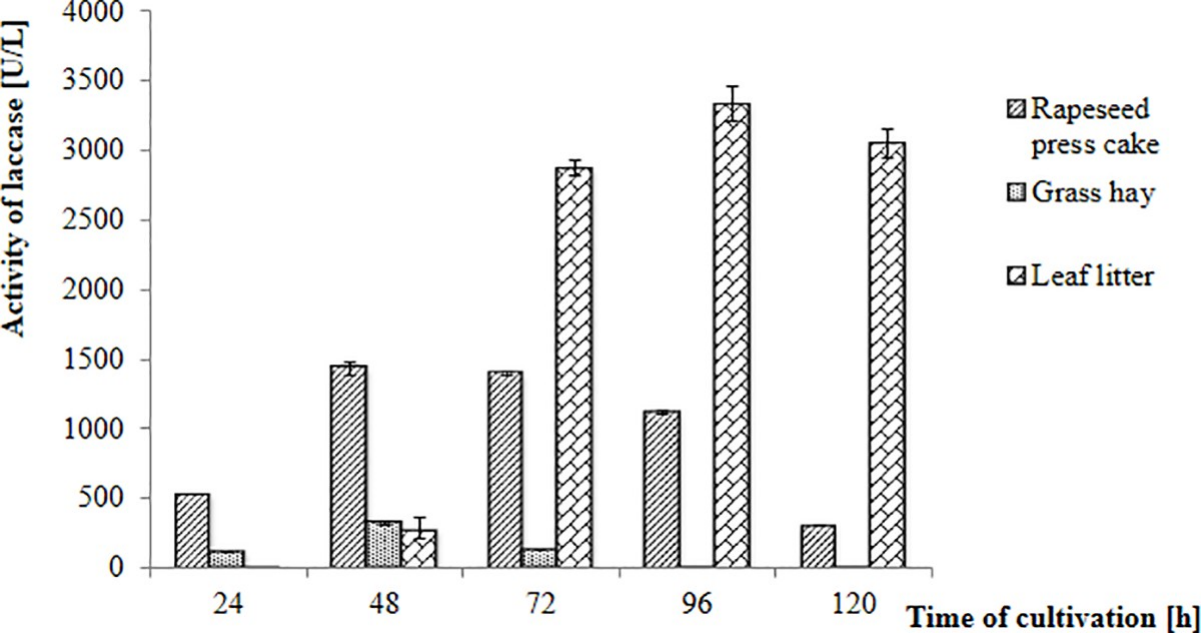
Laccase production by the *Nectriella pironii* IM 6443 strain during the culture in media containing bio-waste material.

Phenolic compounds are natural products of lignin degradation, thus lignocellulosic wastes can be utilized for the enhancement of low-cost laccase production [49, 50]. So far, various lignocellulosic materials, mainly constituting wastes of arable crops, have been used for the production of fungal laccases. For example, lavender straw [51], wheat bran [52], orange peel [53], and sesame oil cake [54] have been utilized as substrates for laccase production by *Pycnoporus cinnabarinus*, *Cyathus bulleri*, *Trametes polyzona*, and *Pleurotus* sp., respectively. In the presented study, extracts of grass hay, rapeseed oil cake, and litter of leaves collected in an urban park in autumn were used to prepare an alternative medium for laccase production. Grass hay and rapeseed oil cake have been previously applied for laccase production by *M*. *roridum* IM 6482 [13], while there have been no studies on the use of naturally fallen leaves as a substrate for fungal laccase production. However, under natural conditions, the decomposition of plant litter is associated with the secretion of extracellular enzymes like laccases whose concentration and activity may change depending on the climate, litter quality, and the decomposer communities [18]. This finding suggests that litter of leaves might serve as a good substrate for enzyme production. According to the results presented in Fig 4, the nature of waste material used to prepare the medium strongly influenced the laccase production by *N*. *pironii* IM 6443. The highest activity of the enzyme was obtained after 96 hours of cultivation in growth medium based on the leaf litter. Laccase activity was found to be 3,330 U/L and was similar to those obtained in media containing copper ions and ferulic acid. To the best of our knowledge, this is the first study to report on laccase production from a *N*. *pironii* strain grown on the extract obtained from leaf litter.

The presented results are promising as they not only indicate an economical method of obtaining laccase, but also demonstrate how to manage the previously unnecessary and decayed bio-waste. Considering the production scale of waste leaves, the possibility of using them in the process seems to be profitable. The studies of Hereźniak [55] and Długoński and Szumański [25] revealed that an estimated 0.8 Mg of leaf bio-waste (fresh matter) can be obtained from one adult urban tree on a yearly basis. By averaging these statistical data, it can be evaluated that about 5.4 Mg of wet leaf litter can be provided annually by adult trees of *A*. *hippocastanum* L. and *P*. *simonii “*Fastigiata” growing in the studied area (the Plac Komuny Paryskiej pocket park).

### Identification of the protein with the laccase activity by mass spectrometry

The extracellular laccase produced on leaf extract medium by *N*. *pironii* was purified using a multistep purification strategy that included ammonium sulfate precipitation, ultrafiltration, ion-exchange chromatography on the HiTrap Q FF column, and a gel filtration step on the HiPrep 16/60 Sephacryl S-200 column. The purity and enzymatic activity of the obtained laccase enzyme was confirmed by the presence of a single band on a zymogram stained with ABTS solution and its molecular weight was found to be around 50 kDa, which is consistent with the typical weight of laccases obtained from white-rot fungi [56].

**Fig 5:**
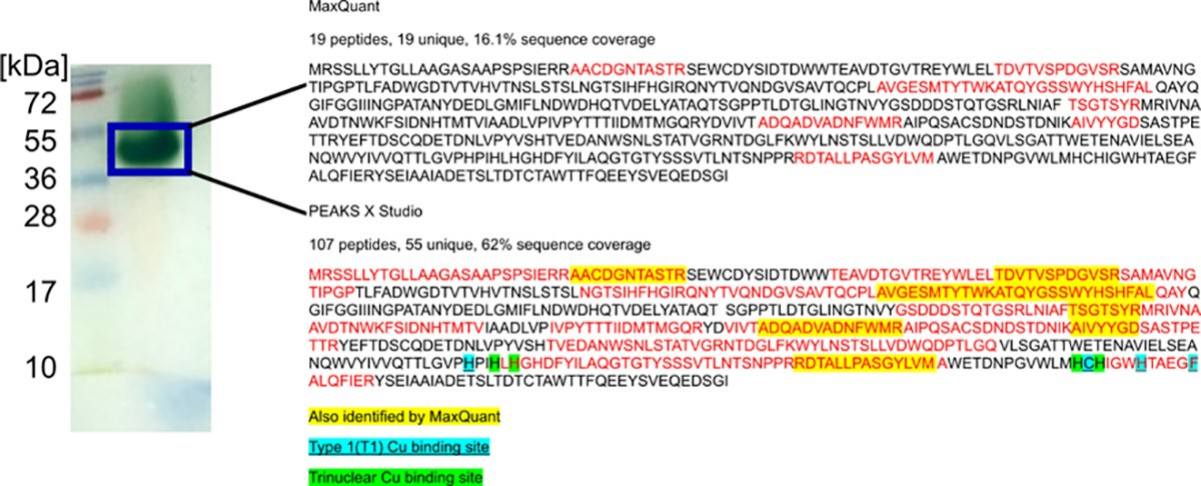
Mass spectrometry identification of the laccase preparations purified from *Nectriella pironii*.

The purified protein possessing the laccase activity was visualized on an in-gel zymogram using ABTS as a substrate, and the ABTS-positive protein band was subsequently subjected to mass spectrometry identification (Fig 5). Searching the MS/MS fragmentation spectra against the initial “multicopper oxidase” database resulted in the identification of XP_018036634.1 multicopper oxidase from *Paraphaeosphaeria sporulosa* as the initial best hit. Since *P*. *sporulosa* is not closely related to *N*. *pironii*, the search was repeated against a secondary database containing FASTA records of proteins showing a significant similarity to the initial identification, but not necessarily annotated as multicopper oxidases. The results suggest that the laccase characterized in the current study belongs to a conserved family of laccases related to Laccase-2 from *Fusarium oxysporum* and Laccase-2 from *Colletotrichum trifolii* (S1 File). Several peptides were matched to the KFA51000.1 protein, a 61,845 Da hypothetical protein from *S*. *chartarum* IBT40293, by using both MaxQuant and PEAKS X Studio software suites. MaxQuant reported 19 unique peptides, whereas PEAKS X Studio reported 55 unique peptides matching the KFA51000.1 protein, with 16.1% and 62% protein sequence coverage, respectively. Importantly, all the regions of protein sequence match covered by MaxQuant were also covered by PEAKS. The KFA51000.1 protein, though being identified as hypothetical, shows high sequence conservation and belongs to the same protein family as the above-mentioned laccases (S1 File). The final identification of the *N*. *pironii* laccase as a protein most closely related to KFA51000.1 is also one of the best possible matches with species phylogeny, as species of *Stachybotrys* are amongst the closest cousins of *Nectriella*.

### Laccase characterization

**Fig 6:**
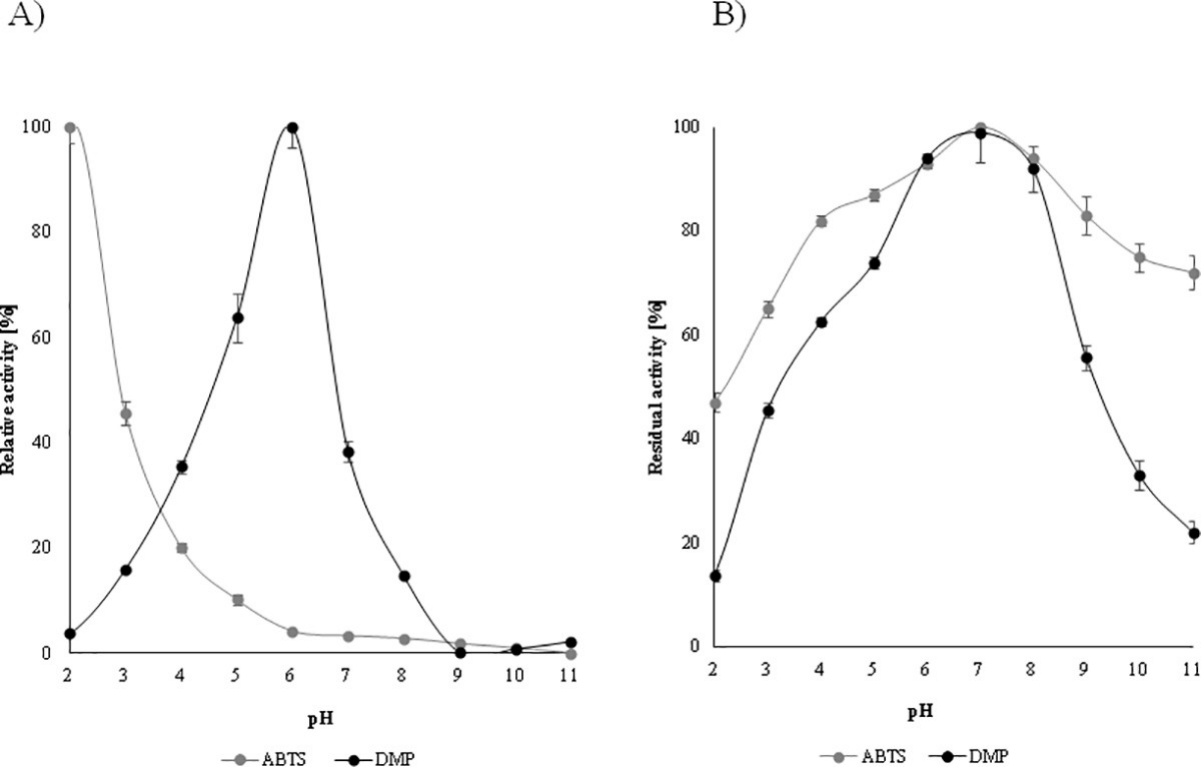
Effect of pH on the activity (A) and stability (B) of the purified laccase with ABTS and 2,6-DMP as substrates.

The effect of the pH on the activity and stability of enzyme is presented in Fig 6. The optimum pH of the *N*. *pironii* IM 6443 laccase depends on the substrate employed. The optimum pH was observed in Mc Ilvaine buffer with pH 2.0 or 6.0 when 10 mM ABTS or DMP were, respectively, used as a substrate. Most fungal laccases are active at acidic pH values and lose activity under alkaline conditions [3]. The differences in pH optima between ABTS and DMP reflect the differences in the oxidation mechanism in various substrates [57]. In general, the catalytic activity of laccase shows a bell-shaped pH profile for the majority of substrates. The pH stability of *N*. *pironii* IM 6443 laccase was determined, and it retained almost 100% activity after 3 h at pH 6.0–8.0 for both the tested substrates. Moreover, it showed more than 50% residual activity after incubation at pH 4.0–9.0 or 3.0–10.6 when DMP or ABTS, respectively, were used. Zhao et al. [58] reported 40% activity of *M*. *verrucaria* NF-05 laccase at pH range 3–7 after 1 h of incubation. The stability in a wide pH range is a beneficial feature in different industrial and biotechnological applications [40]. The pH of industrial wastewater, depending on the process, can differ dramatically, thus supporting wastewater treatment opportunities for the *N*. *pironii* IM 6443 laccase enzyme.

The enzymatic activity of the *N*. *pironii* laccase was found to be completely inactivated at 0.1 mM concentration of sodium azide, DTT, and β-ME (Table 2). Similar results were obtained by Mukhopadhyay and Banerjee [59], who reported a total inhibition of *Lentinus squarrosulus* laccase by 0.1 mM DTT. Xu et al. [57] demonstrated that 12.5 mM concentration of β-ME caused total inhibition due to the reduction of disulfide bonds. The inhibitory effect rose with increasing concentrations of all the studied compounds. Kojic acid at 10 mM concentration caused 99% laccase inhibition, while the presence of urea did not show a significant inhibitory effect at concentrations below 100 mM.

**Table 2:** Effect of inhibitors on laccase activity.

Inhibitor	Concentration [mM]	Inhibition [%]
**SDS**	0.1	89.25 ± 0.12
	1	99.82 ± 0.08
	5	99.93 ± 0.11
**Urea**	50	76.41 ± 0.19
	100	89.14 ± 0.21
	500	94.75 ± 2.56
**EDTA**	1 100	95.60 ± 1.43 99.99 ± 3.71
**NaN**_**3**_	0.1	100.00 ± 7.98
**DTT**	0.1	100.00 ± 5.54
**β—mercaptoethanol**	0.1	99.90 ± 0.17
**Kojic acid**	1 5 10	97.47 ± 0.52 98.33 ± 0.78 99.00 ± 0.09

### Decolorization of synthetic dyes

**Fig 7:**
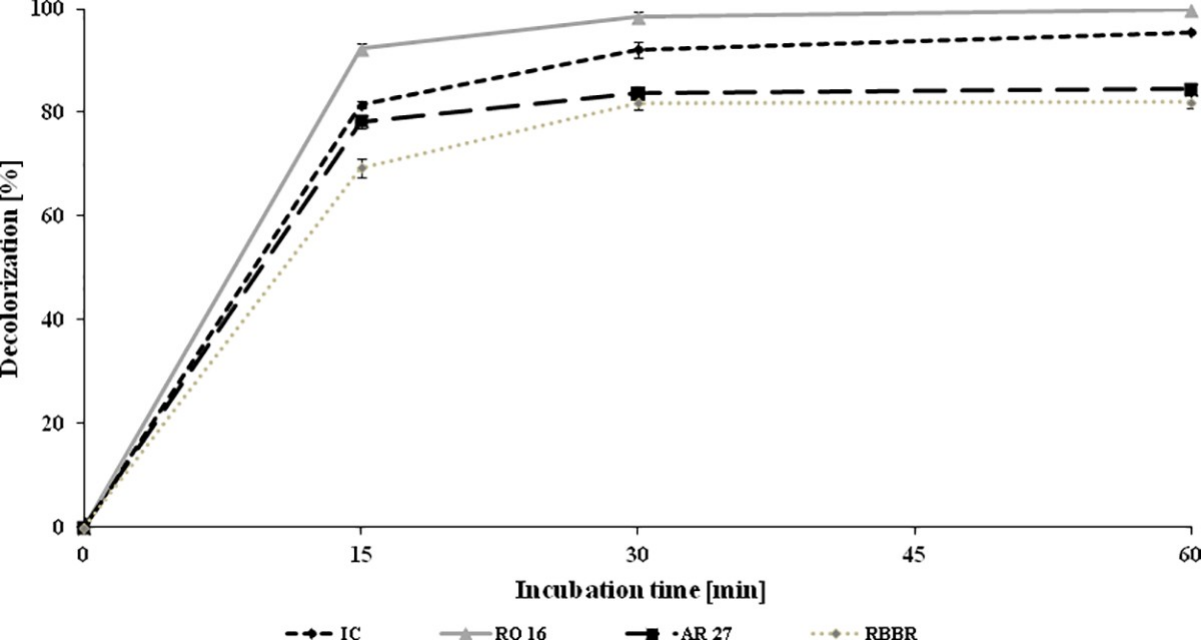
Decolorization of IC, RBBR, RO 16, and AR 27 (25 mg/L) by purified laccase (1 U/mL) at pH 3.0.

The decolorization potential of laccase purified from *N*. *pironii* IM 6443 was evaluated for four dyes belonging to indigoid, anthraquinone, and azo classes. IC, RBBR, RO 16, and AR 27 are widely used as coloring agents in textile, leather, paper, and phenol-formaldehyde resin industries as well as food additives. However, there are increasing numbers of studies reporting about adverse effects of these compounds on the health of animals and humans. For example, since 1976, AR 27 has been banned in the United States by the Food and Drug Administration (FDA) as a suspected carcinogen. Nevertheless, its use is still legal in some countries. Till date, at industrial scale mainly physical and chemical methods have been applied for the removal of IC, RBBR, RO16, and AR 27 from wastewater [60]. However due to their shortcomings, biodegradation of synthetic dyes by different microbes has recently become an area of major scientific interest [61]. In the present study, 1 U/mL of the enzyme was incubated with 25 mg/L of the dye for 60 min. As shown in Fig 7, 69–92% of the dye was decolorized within 15 minutes of incubation with *N*. *pironii* IM 6443 laccase enzyme. Extending the incubation time to 1 hour slightly increased the level of dye decolorization. Similarly, Shobana and Thangam [62] obtained almost complete elimination of RO16 in a culture of *Nocardiopsis alba* isolated from acclimated sludge released from a textile wastewater treatment plant. On the other hand, laccase from the white-rot fungus *Marasmius scorodonius* was found to decolorize 48% and 61% of RO16 and RBBR, respectively, only in the presence of 1-hydroxybenzotriazole as a redox mediator [63]. The potential of bacteria and white rot-fungi has been applied for IC, RBBR, RO 16, and AR 27 decolorization, while ascomycetous fungi and their laccases have not yet been sufficiently explored in this aspect.

## Conclusion

This is the first study to report about the potential use of an ascomycetous fungus *N*. *pironii* IM 6443, isolated from a postindustrial textile green area, as a profitable laccase producer by utilizing extract of leaf litter as substrate. Efficient laccase production was achieved *via* the synergistic action of 1 mM copper sulfate and ferulic acid as well as through an environmentally friendly approach allowing for the management of decayed bio-waste of urban park leaves. After purification, the extracellular enzyme was identified *via* mass spectrometry as a laccase with the highest amino acid sequence similarity to *S*. *chartarum* laccase. The purified laccase exhibited high stability in a wide range of pH values and was found to effectively decolorize the dyes, which makes it a propitious implement for use in different environmental and industrial applications.

## Supporting information

S1 Fig The phylogenetic tree of *Nectriella pironii* IM 6443. (TIF)

S1 File Peptide identification details for MS experiments. (XLSX)

S1 Raw image (TIF)
