# Correction: Laccase activity of the ascomycete fungus *Nectriella pironii* and innovative strategies for its production on leaf litter of an urban park

**DOI:** 10.1371/journal.pone.0233553

**Published:** 2020-05-14

**Authors:** Aleksandra Góralczyk-Bińkowska, Anna Jasińska, Andrzej Długoński, Przemysław Płociński, Jerzy Długoński

There is an error in affiliation 2 for author Andrzej Długoński. The correct affiliation 2 is: Institute of Biological Sciences, Cardinal Stefan Wyszynski University, Warsaw, Warsaw, Poland.
