# Supplementary figures and images for: Laccase activity of the ascomycete fungus Nectriella pironii and innovative strategies for its production on leaf litter of an urban park
Source: PLoS One. 2020 Apr 16;15(4):e0231453. doi: 10.1371/journal.pone.0231453 (PMC7162450; doi:10.1371/journal.pone.0231453)

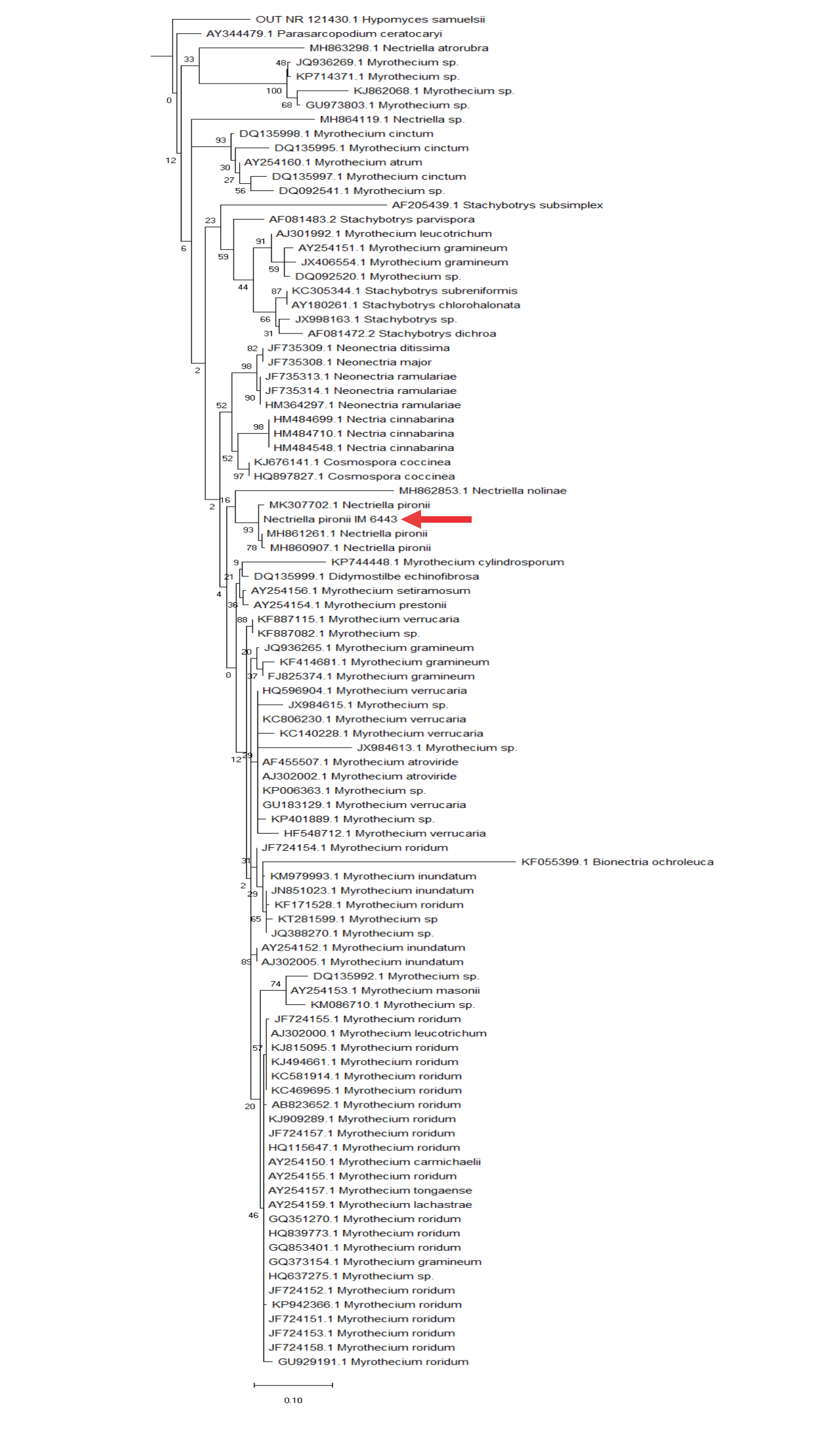

Supplement: S1 Fig — (TIF) [file pone.0231453.s001.tif]

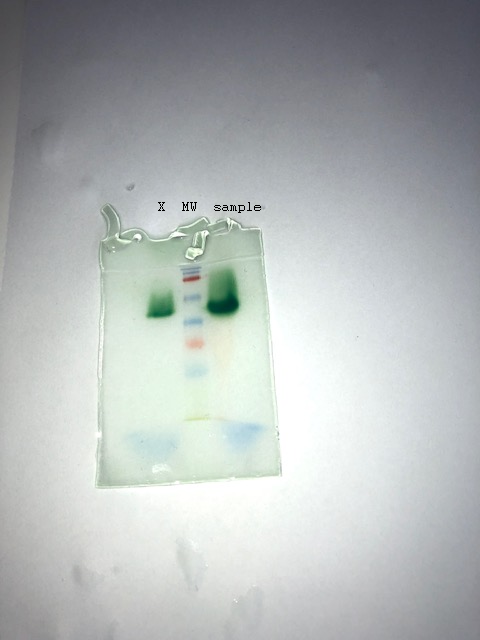

Supplement: S1 Raw image — (TIF) [file pone.0231453.s003.tif]
